# The Cardio-Hepatic Relation in STEMI

**DOI:** 10.3390/jpm11121241

**Published:** 2021-11-23

**Authors:** Lian Bannon, Ilan Merdler, Nir Bar, Lior Lupu, Shmuel Banai, Giris Jacob, Yacov Shacham

**Affiliations:** 1 Department of Gastroenterology and Hepatology, Tel Aviv Sourasky Medical Center, Tel Aviv 64239, Israel; liandhn@gmail.com (L.B.); nirbar7@gmail.com (N.B.); 2 Cardiology Department, Tel Aviv Sourasky Medical Center, Tel Aviv 64239, Israel; Ilanmerdler@gmail.com (I.M.); liorlupu@gmail.com (L.L.); shmuelb@tlvmc.gov.il (S.B.); 3 Internal Medicine F, Tel Aviv Sourasky Medical Center, Tel Aviv 64239, Israel; jacobgi@tlvmc.gov.il

**Keywords:** STEMI, acute heart failure (AHF), cardiac hepatopathy, ACLI, cardio hepatic, acute liver injury, liver enzymes

## Abstract

Background: Hepatic injury secondary to congestive heart failure is well described, however, only limited data exist about the possible impact of acute cardiac dysfunction on the liver. We aimed to explore the possible cardio-hepatic interaction in patients with myocardial infarction. Material and methods: A single-center retrospective cohort study of 1339 ST elevation myocardial infarction (STEMI) patients who underwent primary coronary intervention between June 2012 to June 2019. Echocardiographic examinations were performed to assess left ventricular ejection fraction (LVEF) and central venous pressure (CVP). Patients were stratified into four groups by their LVEF and CVP levels: LVEF ≥ 45%, and CVP ≤ 10 mm/Hg (*n* = 853), LVEF < 45% with CVP ≤ 10 mm/Hg (*n* = 364), EF ≥ 45%, with CVP > 10 mm/Hg (*n* = 61), and LVEF < 45% with CVP > 10 mm/Hg (*n* = 61). Patients were evaluated for baseline and peak liver enzymes including alanine transaminase (AST), alanine aminotransferase (ALT), gamma glutamyl transferase (GGT), alkaline phosphatase (ALP), and bilirubin. Results: Greater severity of cardiac dysfunction was associated with worse elevation of liver enzymes. We found a graded increase in mean levels of maximal ALT, first and maximal ALP, and first and maximal GGT values. Using propensity score matching to estimate the impact of cardiac dysfunction on liver injury, we chose patients with the worst cardiac function parameters: (LVEF < 45% and CVP >10 mm/Hg; *n* = 61) and compared them to matched patients with better cardiac function (*n* = 45). We found a significantly higher level of maximal ALT, first and maximal ALP, and GGT values in the group with the worst cardiac function parameters (*p* < 0.05). Conclusions: Among patients with STEMI, the combination of decreased LVEF and venous congestion was associated with liver enzymes elevation suggesting a possible cardio-hepatic syndrome.

## 1. Introduction

The relation between chronic heart failure and liver injury has been well described [1,2]. A quarter of the cardiac output (CO) goes to the liver by a dual blood supply which consists of the hepatic artery and portal vein [3]. As a result, the liver is more sensitive to hypoperfusion caused by a reduction in CO and decreased hepatic arterial flow. The term cardiac hepatopathy (CH) describes liver damage caused by cardiac disorders. CH consists of congestive hepatopathy and acute cardiogenic liver injury (ACLI) [4]. Congestive hepatopathy usually occurs in chronic heart failure as a result of increased hepatic venous pressure, decreased hepatic blood flow, and a decrease in arterial oxygen saturation [5]. ST elevated myocardial infraction (STEMI) is a major public health concern, associated with high morbidity and mortality rates. Acute cardiac dysfunction caused by STEMI may lead to ACLI due to the combination of rapid reduction in CO and tissue perfusion and passive venous congestion [5,6]. Only limited information is present on the possible cardio-hepatic interaction among patients with STEMI [7,8,9,10,11] We aimed to evaluate the possible cardio-hepatic interaction in a large cohort of STEMI patients undergoing Percutaneous Coronary Intervention (PCI). We hypothesized that the acute cardiac dysfunction (both systolic &diastolic) may result in ACLI, with correlation to the degree of cardiac dysfunction.

## 2. Materials and Methods

A retrospective study was conducted at the Tel Aviv Sourasky Medical Center, a tertiary referral hospital with accessible primary PCI services. We examined a cohort of 2967 patients with STEMI who underwent primary PCI, between June 2012 to June 2019 at the Cardiac Intensive Care Unit (CICU). Males and females above 18 years were included. After excluding patients with chronic liver disease (liver cirrhosis and liver cancer) and excluding those with missing echocardiographic parameters (*n* = 1628), the final cohort included 1339 patients.

### 2.1. Clinical History

Data about patients’ demographics, personal or family history (a first degree relative with cardiovascular disease before the age of 55 for males and 65 for females) of cardiac diseases, comorbidity, treatment, laboratory results, and echocardiography parameters were collected from hospital records.

STEMI was diagnosed by standard clinical, electrocardiographic, and laboratory parameters according to accepted guidelines [12]. Based on this guideline, hemodynamic instability was defined as a need for intravenous inotropes or intra-aortic balloon counter pulsation insertion.

### 2.2. Echocardiography Parameters

All patients underwent echocardiographic examination within up to 3 days of admission, using a Philips IE-33 equipped with s5-1 transducers (Philips Healthcare, Andover, MA, USA) and a GE Vivid 7 model equipped with M4S transducer. Echocardiography parameters were retrieved from medical records. LVEF was calculated by the biplane methods of disks (modified Simpson’s rule). We used 45% as the cutoff for low or non-low values. Central venous pressure (CVP) was evaluated by assessing the IVC inspiratory and expiratory diameter and the percentage of IVC collapse. 

Normal CVP of 3 mm/Hg (range 0–5 mmHg) was assumed when IVC diameter < 2.1 cm that collapsed above 50% with a sniff; high CVP of 15 mmHg (range 10–20 mmHg) was assumed when IVC diameter > 2.1 cm collapsed at less than 50% with sniff. In case the IVC diameter and collapse did not fit these rules, a hepatic flow pattern was used to assess the CVP. Normal CVP (3 mmHg, range 0–5 mmHg) was assumed if systolic wave velocity was greater than the diastolic wave velocity (systolic predominance). Finding of systolic predominance loss was suggested at high CVP pressure (15 mmHg, range 10–20 mmHg). In uncertain cases, CVP was counted as intermediate (8 mmHg, range 6–10). In this study, we used a CVP cutoff of 10 mm/Hg to define normal or high values [13,14,15,16]. 

Patients were divided into four groups by their LVEF and CVP levels: LVEF ≥ 45%, and CVP ≤ 10 mm/Hg (*n* = 853), LVEF < 45 with CVP ≤ 10 mm/Hg (*n* = 364), EF ≥ 45%, with CVP > 10 mm/Hg (*n* = 61), and LVEF < 45% with CVP > 10 mm/Hg (*n* = 61). We defined patients with the worst cardiac function as those with EF > 45% and CVP >10 mm/Hg. 

We used propensity score matching to compare liver enzymes elevation in this group to patients with better cardiac function (the other 3 groups). 

### 2.3. Blood Tests

Blood samples were obtained at baseline (admission day) and then daily until discharged, patients had a complete blood count, PT, PTT, INR, cardiac biomarkers (troponin), and liver enzymes serially measured. The liver enzymes included: alanine transaminase (ALT) and aspartate transaminase (AST), gamma-glutamyl transferase (GGT), serum total bilirubin, and alkaline phosphatase (ALP). 

We recorded the first liver enzyme values defined as the baseline level taken on admission day. Maximal values were defined as the highest documented levels of liver enzymes which were measured from sequential blood tests through hospitalization. 

First and maximal values were used for the analysis.

The local institutional ethics committee (Helsinki committee Tel Aviv medical center, Tel Aviv, Israel) approved this present study protocol (IRB num TLV-16-224).

### 2.4. Statistics

Categorical variables were reported as numbers and percentages and compared using Pearson’s Chi test or Fisher’s exact test. Continuous variables were reported as mean ± standard deviation (SD) or as median ± IQR and compared using *t*-test or Mann–Whitney test and were tested for normal distribution using the Shapiro-Wilk test, histograms, and Q–Q Plots. Odds ratios (OR) and 95% confidence intervals (CI) were reported for the main study outcomes measures. A two-tailed p value less than 0.05 was considered statistically significant. We used propensity matching to compare groups. To isolate the possible impact of cardiac dysfunction on liver injury, we used propensity score matching to compare patients with the worst cardiac function parameters [EF > 45% and CVP > 10 mm/Hg], and patients with the same demographics and clinical characteristics but better cardiac function. Logistic regression was used to calculate the propensity score. Age, gender, smoking status, time to the emergency room (time to the ER), DM status, hyperlipidemia and HTN were included in the logistic regression. Patients were matched according to the propensity score and an absolute difference up to 5% was considered as acceptable. An absolute standardized difference was used to evaluate differences between the matched groups before and after matching. Mirrored histogram was used to describe the sampling method of the matched groups according to the propensity score. The paired sample *t*-test, Wilcoxon test or McNemar’s test were used to compare the matched groups. All statistical analyses were performed with SPSS (IBM SPSS Statistics for Windows, Version 25.0, released 2013).

## 3. Results

A total of 1339 patients were included in the present study, mean age was 61 ± 13 years, 1082 (80.8%) of them were men. Patients were divided into four groups by their LVEF and CVP levels: LVEF ≥ 45%, and CVP ≤ 10 mm/Hg (*n* = 853), LVEF < 45% with CVP ≤ 10 mm/Hg (*n* = 364), EF ≥ 45%, with CVP > 10 mm/Hg (*n* = 61), and LVEF < 45% with CVP > 10 mm/Hg (*n* = 61) (Table 1).

**Figure 1:**
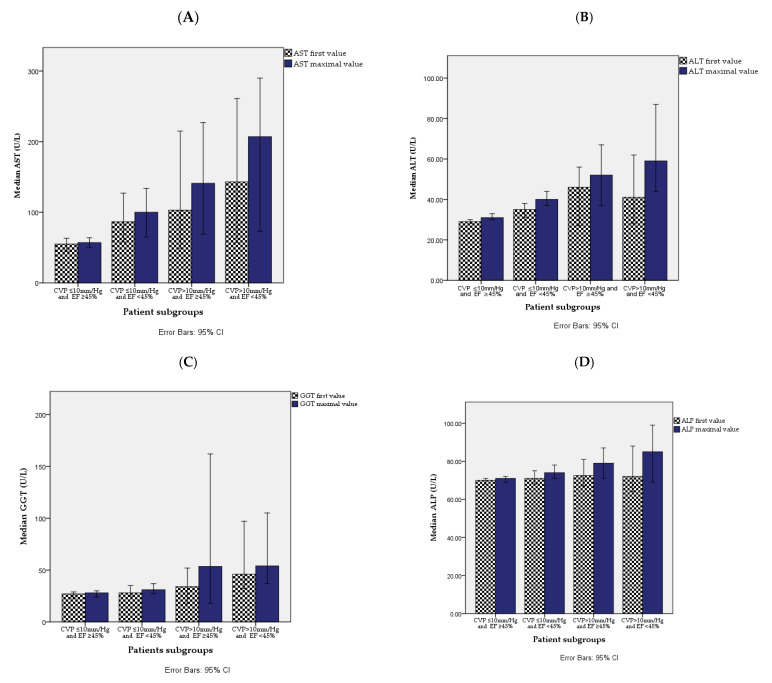
Median levels of baseline and peak liver enzymes in four groups. (**A**) AST first and maximal values, (**B**) ALT first and maximal values, (**C**) GGT first and maximal values and (**D**) ALP first and maximal value.

A higher median of first and maximal ALT, AST, GGT, serum total bilirubin, and of maximal ALP was found in the worst cardiac dysfunction. The levels of median C- reactive protein (CRP) and creatine phosphor kinase (CPK) were also significantly higher in patients with the worst cardiac dysfunction (Table 2 and Figure 1).

To isolate the possible impact of cardiac dysfunction on liver injury, we used propensity score matching to compare patients with the worst cardiac function parameters [EF > 45% and CVP >10 mm/Hg, (*n* = 61)] and patients with the same demographics and clinical characteristics but better cardiac function (*n* = 45). 

Patients were matched for baseline cohort characteristics with a significant level of *p* value < 0.05 (Table 3 and Table S1).

## 4. Discussion

In the present study of STEMI patients, we showed that greater severity of cardiac dysfunction was associated with graded elevation of liver enzymes. When we focused on patients with the worst cardiac function, we found significantly higher levels of both hepatocellular and cholestatic enzymes compared to the rest of the cohort, even after propensity score matching.

### 4.1. Cardio-Hepatic Interaction

The liver receives 25% of the total CO, although it accounts for only 2.5% of the total body weight [3]. The hepatic arteries blood, which is characterized with high pressure and is well oxygenation, supplies only about one fifth to one third of the total liver blood flow. The approximate other two thirds supplied by the low pressure, less oxygenate blood of the portal venous. [17] The hepatic artery autoregulation caused by hepatic arterial buffer response may compensate for a 25–60% reduction in portal blood flow. On the contrary, in case of a decrease in hepatic blood flow, the portal vein depends on the hepatic-portal venous pressure gradient and on the mesenteric circulation. The dual liver vasculature protects the liver from hypoperfusion as it is particularly sensitive to perfusion changes [5]. The cardio-hepatic relationship initially has been described in 1833 by Kiernan, who coined the term of a post-mortem congested “nutmeg” liver in patients with heart failure [18]. In 1951, Sherlock et al., described the findings of hepatic cell necrosis in every form of heart failure. He claimed that anoxia causes the degeneration of central liver cells and the dilatation of sinusoids. Centrilobular necrosis may be caused by increased pressure in the hepatic veins [19]. Chronic right heart failure may lead to CH, which is mainly caused by the passive congestion of the liver and results in elevated CVP [20]. Different etiologies for right-sided heart failure may precipitate CH, including constrictive pericarditis, mitral stenosis, tricuspid regurgitation, cardiomyopathy, and cor pulmonale. There are also congenital heart diseases related to elevated CVP and passive congestion as atrial and ventricular septal defect, Ebstein anomaly, etc. [6]. Patients with abnormal liver enzymes and right-side heart failure or any cause of elevated central pressure should be suspected of CH. Coexistence of the left and right-side heart failure results in reduced CO and arterial perfusion and venous congestion, worsening liver injury [4,21].

### 4.2. Acute Cardiogenic Liver Injury

Due to the liver-enhanced sensitivity to perfusion changes, acute circulatory changes, related to acute decompensated heart failure; acute MI; myocarditis; massive PE, or cardiogenic shock, might result in ACLI [22]. The pathogenesis of ACLI includes a decrease in CO and tissue perfusion, hypoxemia, and passive venous congestion. The histologic appearance of ACLI is characterized by necrosis of pericentral zone 3 hepatocytes, which received poorly oxygenated blood compared with zones 1 and 2. ACLI is usually asymptomatic at the beginning, but 2–24 h after acute insult, patients may suffer from nausea, vomiting, right upper quadrant pain, and apathy. In rare cases, mental confusion, jaundice, and even hepatic coma may develop [17,18,19,20,21]. The common laboratory findings in ACLI are elevated transaminases and LDH, which reach their peak in 1–3 days after the acute insult, and in case of improvement, return to the normal limits within 7–10 days after [4].

Only a few and limited studies evaluated the cardio-hepatic relation in acute cardiac injury, as occur in patients with STEMI. Ming et al. studied STEMI patients with no preexisting liver disease, who underwent primary PCI. They showed that serum transaminases ≥95th percentage were associated with increased incidence of short- and long-term all-cause mortality [7]. A recent study on patients with STEMI who underwent primary PCI demonstrated that combined ALKP and hypoxic liver injury (HLI) in addition was associated with poor clinical outcomes [8]. Another retrospective study of STEMI patients who underwent primary PCI with no preexisting liver disease, found the presence of HLI correlated with HFREF and associated with poor survival [9]. Another study showed that increased ALK-P was associated with higher in-hospital event rates in patients with STEMI who underwent primary PCI [10]. On the other hand, in patients with STEMI, the presence of elevated GGT is associated with long-term mortality after 30 days after PCI [11]. Post hoc analysis of survival showed that liver function impairment was associated with a 50% increase in the 180-day mortality rate in patients with acute decompensated heart failure and required inotrope treatment. Authors suggested the high level of ALK-P and/or transaminases apparently reflected the severity of the underline HF [23]. A possible mechanism for liver injury is elevated hydrostatic pressure related to liver sinusoids which might cause bile canaliculi and ductulus compression. In addition, hepatic cytolysis may be secondary to the hypoperfusion/hypo-oxygenation of the centrilobular region. A 46–76% liver function abnormality was reported in patients with acute heart failure, suggesting to measure the baseline liver function test of patients present with acute decompensated heart failure and, in addition, to consider adding it to future HF guidelines [24].

### 4.3. The Impact of Liver Injury on the Heart

Cirrhotic cardiomyopathy is the concomitant cardiac dysfunction in patients with cirrhosis and having hyperdynamic systemic circulation, characterized by an increased heart rate and cardiac output, and a decreased arterial blood pressure and systemic vascular resistance [25]. In addition, there is an increased cardiac pre-load and right atrial pressure, cardiac contractile impairment, diastolic dysfunction, and electrophysiological disorders such as prolonged Q- T interval. Cardiac morphometric changes are associated with the severity of liver failure regardless of liver cirrhosis etiology [26]. The prevalent of diastolic dysfunction in cirrhosis may be up to 50% and correlate with severity of cirrhosis, complication, and death [26,27,28].

Patients with acute liver failure (ALF) caused by different etiologies such as viral hepatitis and acetaminophen-induced, may have an elevation of troponin I levels which reflect myocardial cell damage. The exact mechanism of that unrecognized cardiac injury and potential of reversibility is not clear. The subclinical myocardial injury in patients with ALF is associated with higher morbidity and mortality rates [29]. Critically ill noncardiac patients may develop myocardial oxygen supply and demand imbalance, which may result in a higher level of troponin I and associated with elevation of Interleukin 6, Tumor necrosis factor-alpha (TNF-α), and CRP. TNF-α is known to be able to depress myocardial function and induce the apoptosis of cardiomyocytes and may lead to reduced coronary artery flow and EF [29,30]. Our findings point to the importance of the cardio-hepatic interaction. In patients with an acute cardiac insult (e.g., STEMI), sequential liver function tests taken throughout the hospitalization may alert clinicians to liver enzyme abnormalities which were associated with poorer cardiac presentation (low cardiac output and/or signs of venous congestion). In addition to the possible relation to patients’ outcomes, a clinical decision of withholding hepatotoxic drugs earlier should be considered [31,32]. This offers a non-invasive and simple way to risk stratify patients, which should be further validated in prospective studies.

### 4.4. Limitations

- One main limitation is the retrospective design of this study.
- Data about patients’ structural diseases, especially of NAFLD/NASH are most likely underestimated by using ICD-9. We can assume by patients’ chronic diseases as HTN, DM-2, and dyslipidemia that many patients may have liver steatosis, which makes them more vulnerable to liver injury due to cardiac dysfunction.
- Liver enzymes were evaluated only at the admission period and there is no long-term follow up on cohort patients, thus we cannot evaluate patient liver condition nor prognosis.
- Estimation of CVP was based on rough means rather than invasive hemodynamic data, which may have effects on the accuracy of measurements and data analysis.
- Patients with worse cardiac function were more likely to have impaired hemodynamics and be mechanically ventilated. This by itself may have contributed to the worsening of liver function and might negate the effect of the propensity score matching. Our database does not include information on patients’ body mass index. Body mass index is a known and important confounder, as it may impact liver enzymes via increased prevalence of steatosis in high body mass index patients and also impacts outcomes in STEMI.

## 5. Conclusions

We found a significant association between the severity of cardiac dysfunction and liver function in patients with STEMI. Among patients with STEMI, the combination of decreased CO and venous congestion may result in cardio-hepatic syndrome. Further studies are needed to assess the cardio-hepatic relation in acute heart failure.

## Figures and Tables

**Table 1:** Patients’ baseline criteria based on cardiac function.

	All patients, *n* = 1339	CVP ≤ 10 mm/Hg and EF ≥ 45% *n* = 853	CVP ≤ 10 mm/Hg and EF < 45% *n* = 364	CVP > 10 mm/Hg and EF ≥ 45% *n* = 61	CVP > 10 mm/Hg and EF < 45% *n* = 61	*p* Value
Age (years), median, IQR	61 (53–70)	60 (51–67)	62 (53–73)	65 (56–72)	72 (63–83)	0.000
Male, *n* (%)	1082 (80.8)	708 (83)	281 (77.2)	45 (73.8)	48 (78.6)	0.050
Family history *n* (%)	303 (22.6)	210 (24.6)	80 (22)	11 (18)	2 (3.2)	0.001
Smoker *n* (%)	695 (51.9)	449 (52.6)	189 (52)	34 (55.8)	23 (37.8)	0.140
Diabetes mellitus, *n* (%)	320 (23.9)	191 (22.4)	90 (24.8)	22 (36)	17 (27.8)	0.082
Hyperlipidemia *n* (%)	617 (46)	401 (47)	158 (43.4)	34 (55.8)	24 (39.4)	0.191
Hypertention *n* (%)	589 (43.9)	350 (41)	168 (46.2)	35 (57.4)	36 (59)	0.003
EGFR < 60 *n* (%)	257 (19.19)	133 (15.6)	87 (24)	10 (16.4)	27 (44.2)	0.000
EGFR median IQR	75 (60–89)	76 (63–92)	72 (57–87)	71 (58–89)	57 (44–74)	0.000
Acute kidney injury *n* (%)	154 (11.5)	49 (5.8)	60 (16.4)	16 (26.2)	29 (47.6)	0.000
Mortality in 30days *n* (%)	33 (2.46)	4 (0.4)	12 (3.2)	3 (5)	14 (23)	0.000
IABC- inotropes *n* (%)	49 (3.66)	9 (1)	18 (5)	8 (13.2)	14 (23)	0.000
In hospital CABG *n* (%)	30 (2.24)	10 (1.2)	12 (3.2)	1 (1.6)	7 (11.4)	0.000
Mechanical Ventilation *n* (%)	64 (4.78)	19 (2.2)	24 (6.6)	6 (9.8)	15 (24.6)	0.000
Heart Failure *n* (%)	152 (11.35)	39 (4.6)	75 (20.6)	15 (24.6)	23 (37.8)	0.000
Bradicardia *n* (%)	62 (4.63)	37 (4.4)	9 (2.4)	11 (18)	5 (8.2)	0.000
VT/VF *n* (%)	121 (9.04)	54 (6.4)	42 (11.6)	6 (9.8)	19 (31.2)	0.000
AF *n* (%)	66 (4.93)	34 (4)	16 (4.4)	9 (14.8)	7 (11.4)	0.000
Stent thrombosis (%)	65 (4.85)	36 (4.2)	23 (6.4)	2 (3.2)	4 (6.6)	0.372
Bleeding *n* (%)	68 (5.08)	33 (3.8)	26 (7.2)	3 (5)	6 (9.8)	0.034
Past MI *n* (%)	212 (15.83)	117 (13.8)	74 (20.4)	8 (13.2)	13 (21.4)	0.018
No CAD *n* (%)	2 (0.15)	2 (0.2)	0 (0)	0 (0)	0 (0)	0.004
1 CAD *n* (%)	559 (41.75)	365 (42.8)	152 (41.8)	21 (34.4)	21 (34.4)	0.004
2 CAD *n* (%)	380 (28.38)	267 (31.4)	87 (24)	15 (24.6)	11 (18)	0.004
3 CAD *n* (%)	388 (28.98)	217 (25.4)	119 (32.6)	25 (41)	27 (44.2)	0.004
Contrast volume, median IQR	123 (89–148)	136 (114–169)	150 (118–189)	137 (108–178)	153 (114–170)	0.244
Time to ER, median IQR	120 (60–400)	120 (60–300)	120 (60–480)	132 (60–690)	120 (60–1200)	0.067
Door To Baloon, median IQR	45 (30–60)	45 (30–60)	50 (30–60)	45 (72–30)	50 (30–68)	0.005
Time to reperfusion, median IQR	180 (120–510)	160 (105–360)	180 (110–573)	210 (130–771)	180 (118–870)	0.028

EGFR = Estimated glomerular filtration rate. IABC = Intra-aortic balloon counterpulsation. CABG = coronary artery bypass graft. Vt = Ventricular tachycardia. VF = Ventricular fibrillation. AF = Atrial fibrillation. MI = Myocardial infarction. CAD = Coronary artery disease. ER = Emergency room. Family history refers to family history of cardiac diseases.

**Table 2:** Patients’ baseline and peak liver function tests as well as cardiac biomarkers in each subgroup.Using propensity score matching, we found significantly higher levels of ALT, ALKP and GGT in the group with the worst cardiac function parameters compared to controls. Level of AST was also higher in that group, although not significantly.

	All Patients, *n* = 1339	CVP ≤ 10 mm/Hg and EF ≥ 45% *n* = 853	CVP ≤ 10 mm/Hg and EF < 45% *n* = 364	CVP > 10 mm/Hg and EF ≥ 45% *n* = 61	CVP > 10 mm/Hg and EF < 45% *n* = 61	*p* Value
ALT first, median (U/L) IQR	29 (20–45)	29 (21–44)	35 (23–54)	46 (21–73)	41 (26–88)	0.000
ALT max median (U/L) IQR	33 (22–53)	31 (22–47)	40 (27–64)	52 (25–82)	59 (31–107)	0.000
AST first median (U/L) IQR	74 (35–157)	55 (31–120)	86 (40–199)	103 (43–222)	143 (46–290)	0.000
AST max median (U/L) IQR	78 (38–175)	57 (33–121)	100 (44–229)	141 (54–272)	207 (56–493)	0.000
Total bilirubin—first median (U/L) IQR	0.5 (0.4–0.7)	0.5 (0.4–0.7)	0.5 (0.4–0.7)	0.5 (0.4–0.8)	0.6 (0.4–0.9)	0.000
Total bilirubin—max median (U/L) IQR	0.58 (0.4–0.8)	0.5 (0.4–0.72)	0.58 (0.4–0.8)	0.6 (0.48–1)	0.6 (0.4–0.9)	0.000
Bilirubin indirect—first median (U/L) IQR	0.4 (0.2 – 0.5)	0.4 (0.3–0.5)	0.4 (0.3 – 0.5)	0.4 (0.2–0.7)	0.3 (0.2–0.6)	0.638
Bilirubin indirect—max median (U/L) IQR	0.4 (0.3–0.5)	0.4 (0.3–0.5)	0.4 (0.3–0.6)	0.4 (0.2–0.7)	0.3 (0.2–0.6)	0.559
Bilirubin direct—first median (U/L) IQR	0.2 (0.1–0.3)	0.2 (0.1–0.3)	0.2 (0.1–0.3)	0.2 (0.1–0.3)	0.25 (0.14–0.4)	0.143
Bilirubin direct—max median (U/L) IQR	0.2 (0.1–0.3)	0.2 (0.1–0.3)	0.2 (0.1–0.3)	0.3 (0.1–0.5)	0.3 (0.1–0.5)	0.026
ALP first median (U/L) IQR	68 (56–82)	70 (58–82)	71 (58–86)	72 (58–87)	72 (60–98)	0.273
ALP max median (U/L) IQR	70 (57–84)	71 (58–84)	74 (61–90)	79 (59–91)	85 (62–115)	0.000
GGT first median (U/L) IQR	28 (18–50)	27 (17–42)	28 (17–55)	34 (17–63)	46 (25–112)	0.005
GGT max median (U/L) IQR	30 (19–52)	28 (17–46)	31 (18–58)	53 (17–162)	54 (30–123)	0.001
CRP, median (mg/L) IQR	4 (1–12)	4 (1–9)	5 (2–13)	7 (2–17)	8 (3–25)	0.000
CRP-max, median (mg/L) IQR	21 (9–61)	10 (4–27)	33 (10–116)	56 (10–139)	78 (26–168)	0.000
CPK (peak), median (U/L) IQR	760 (281–1664)	723 (312–1443)	1302 (500–2762)	839 (371–1672)	1112 (399–2682)	0.000
Troponin first median (mg/dL) IQR	13 (0.2–333)	0.11 (0.02–1.58)	0.4 (0.07–4.3)	2 (0.06–13)	1.2 (0.1–13)	0.000
Troponin max median (mg/dL) IQR	145 (6–42833)	21 (5–60)	50 (15–163)	41 (18–80)	65 (15–165)	0.000

**Table 3:** Propensity score matching of liver enzymes for the group of worst cardiac function vs. controls.

	CVP > 10 mm/Hg and EF < 45% (*n* = 61)	All Others (*n* = 45)	*p* Value
ALT first, mean (U/L), Std	78.6 ± 18.5	49 ± 26	0.08
ALT max, mean (U/L), Std	211.6 ± 72.5	49 ± 27	0.01
AST first, mean (U/L), Std	200.5 ± 43.5	186.5 ± 106.5	0.57
AST max, mean (U/L), Std	314.5 ± 85.9	195.5 ± 97.5	0.62
ALK-P first, mean (U/L), Std	88.3 ± 7.4	64 ± 4	0.03
ALK-P max, mean (U/L), Std	122.5 ± 13.4	64 ± 4	0.01
GGT first, mean (U/L), Std	87.6 ± 16.8	13.5 ± 1.5	0.023
GGT max, mean (U/L), Std	139.2 ± 43.9	13.5 ± 1.5	0.023

## Supplementary Materials

The following are available online at https://www.mdpi.com/article/10.3390/jpm11121241/s1, Table S1: Comparison of baseline criteria of patients with the worst cardiac function parameters [EF > 45% and CVP > 10 mm/Hg, (*n* = 61)] and 45 patients with the same demographics and clinical characteristics but better cardiac function (*n* = 45).

## References

[1] Fouad Y.M., Yehia R. (2014). Hepato-cardiac disorders. World J. Hepatol..

[2] Alvarez A.M., Mukherjee D. (2011). Liver Abnormalities in Cardiac Diseases and Heart Failure. Int. J. Angiol..

[3] Lautt W.W., Greenway C.V. (1987). Conceptual review of the hepatic vascular bed. Hepatology.

[4] Cagli K., Başar F.N., Tok D., Turak O., Başar Ö., Çağlı K. (2020). How to interpret liver function tests in heart failure patients?. Turk. J. Gastroenterol..

[5] Hilscher M., Sanchez W. (2016). Congestive hepatopathy. Clin. Liver Dis..

[6] Asrani S., Asrani N.S., Freese D.K., Phillips S.D., Warnes C.A., Heimbach J., Kamath P.S. (2012). Congenital heart disease and the liver. Hepatology.

[7] Gao M., Cheng Y., Zheng Y., Zhang W., Wang L., Qin L. (2017). Association of serum transaminases with short-and long-term out-comes in patients with ST-elevation myocardial infarction undergoing primary percutaneous coronary intervention. BMC Cardiovasc. Disord..

[8] Oh P.C., Eom Y.S., Moon J., Jang H.-J., Kim T.-H., Suh J., Kong M.G., Park S.-D., Kwon S.W., Choe J.Y. (2020). Prognostic impact of the combination of serum transaminase and alkaline phosphatase determined in the emergency room in patients with ST-segment elevation myocardial infarction undergoing primary percutaneous coronary intervention. PLoS ONE.

[9] Moon J., Kang W., Oh P.C., Seo S.Y., Lee K., Han S.H., Ahn T., Shin E. (2014). Serum transaminase determined in the emergency room predicts outcomes in patients with acute ST-segment elevation myocardial infarction who undergo primary percutaneous coronary intervention. Int. J. Cardiol..

[10] Huseynov A., Baumann S., Becher T., Koepp J., Lang S., Jabbour C., Behnes M., Borggrefe M., Akin I. (2016). Liver and cholestatic pa-rameters as prognostic biomarkers of in-hospital MACE in patients with STEMI. Eur. J. Clin. Investig..

[11] Kim J.G., Chang K., Choo E.H., Lee J.-M., Seung K.-B. (2018). Serum gamma-glutamyl transferase is a predictor of mortality in patients with acute myocardial infarction. Medicine.

[12] O’gara P.T., Kushner F.G., Ascheim D.D., Casey D.E., Chung M.K., De Lemos J.A., Ettinger S.M., Fang J.C., Fesmire F.M., Franklin B.A. (2013). 2013 ACCF/AHA Guideline for the Management of ST-Elevation Myocardial Infarction: Executive Summary: A Report of the American College of Cardiology Foundation/American Heart Association Task Force on Practice Guidelines. Am. Coll. Cardiol..

[13] Clemmensen T.S., Eiskjær H., Mølgaard H., Larsen A.H., Soerensen J., Andersen N.F., Tolbod L.P., Harms H.J., Poulsen S.H. (2018). Abnor-mal coronary flow velocity reserve and decreased myocardial contractile reserve are main factors in relation to physical exer-cise capacity in cardiac amyloidosis. J. Am. Soc. Echocardiogr..

[14] Lang R.M., Bierig M., Devereux R.B., Flachskampf F.A., Foster E., Pellikka P.A., Picard M., Roman M.J., Seward J., Shanewise J.S. (2005). Recommendations for Chamber Quantification: A Report from the American Society of Echocardiography’s Guidelines and Standards Committee and the Chamber Quantification Writing Group, Developed in Conjunction with the European Association of Echocardiography, a Branch of the European Society of Cardiology. J. Am. Soc. Echocardiogr..

[15] Nagueh S.F., Kopelen H.A., Zoghbi W.A. (1996). Relation of Mean Right Atrial Pressure to Echocardiographic and Doppler Parameters of Right Atrial and Right Ventricular Function. Circle.

[16] Wiwatworapan W., Ratanajaratroj N., Sookananchai B. (2012). Correlation between inferior vena cava diameter and central venous pressure in critically ill patients. J. Med. Assoc. Thail..

[17] Lautt W.W. (2009). Hepatic circulation: Physiology and pathophysiology. Colloquium Series on Integrated Systems Physiology: From Molecule to Function.

[18] Kiernan F. (1833). XXIX. The anatomy and physiology of the liver. Philos. Trans. R. Soc. Lond..

[19] Sherlock S. (1951). The Liver in Heart Failure Relation of Anatomical, Functional, and Circulatory Changes. Heart.

[20] Weisberg I.S., Jacobson I.M. (2011). Cardiovascular Diseases and the Liver. Clin. Liver Dis..

[21] Naschitz J.E., Slobodin G., Lewis R.J., Zuckerman E., Yeshurun D. (2000). Heart diseases affecting the liver and liver diseases affecting the heart. Am. Hear. J..

[22] Samsky M.D., Patel C.B., DeWald T.A., Smith A.D., Felker G.M., Rogers J.G., Hernandez A.F. (2013). Cardiohepatic interactions in heart failure: An overview and clinical implications. J. Am. Coll. Cardiol..

[23] Nikolaou M., Parissis J., Yilmaz M.B., Seronde M.-F., Kivikko M., Laribi S., Paugam-Burtz C., Cai D., Pohjanjousi P., Laterre P.-F. (2013). Liver function abnormalities, clinical profile, and outcome in acute decompensated heart failure. Eur. Heart J..

[24] Poelzl G., Ess M., Von der Heidt A., Rudnicki M., Frick M., Ulmer H. (2013). Concomitant renal and hepatic dysfunctions in chronic heart failure: Clinical implications and prognostic significance. Eur. J. Intern. Med..

[25] Møller S., Henriksen J.H. (2002). Cirrhotic cardiomyopathy: A pathophysiological review of circulatory dysfunction in liver disease. Heart.

[26] Møller S., Bendtsen F. (2018). The pathophysiology of arterial vasodilatation and hyperdynamic circulation in cirrhosis. Liver Int..

[27] Sampaio F., Pimenta J., Bettencourt N., Fontes-Carvalho R., Silva A.P., Valente J., Bettencourt P., Fraga J., Gama V. (2013). Systolic and diastolic dysfunction in cirrhosis: A tissue-Doppler and speckle tracking echocardiography study. Liver Int..

[28] Ruíz-Del-Árbol L., Achécar L., Serradilla R., Rodríguez-Gandía M.Á., Rivero M., Garrido E., Natcher J.J. (2013). Diastolic dysfunction is a predictor of poor outcomes in patients with cirrhosis, portal hypertension, and a normal creatinine. Hepatology.

[29] Parekh N.K., Hynan L.S., De Lemos J., Lee W.M., Acute Liver Failure Study Group (2007). Elevated troponin I levels in acute liver fail-ure: Is myocardial injury an integral part of acute liver failure?. Hepatology.

[30] Wu T.T., Yuan A., Chen C.Y., Chen W.J., Luh K.T., Kuo S.H., Lin F.Y., Yang P.C. (2004). Cardiac troponin I levels are a risk factor for mor-tality and multiple organ failure in noncardiac critically ill patients and have an additive effect to the APACHE II score in outcome prediction. Shock.

[31] Lofthus D.M., Stevens S.R., Armstrong P.W., Granger C.B., Mahaffey K.W. (2012). Pattern of liver enzyme elevations in acute ST-elevation myocardial infarction. Coron. Artery Dis..

[32] Lazzeri C., Valente S., Boddi M., Mecarocci V., Chiostri M., Gensini G.F. (2014). Clinical and prognostic significance of increased liver en-zymes in ST-elevation myocardial infarction. Int. J. Cardiol..

