# Supplementary material for: The Cardio-Hepatic Relation in STEMI
Source: J Pers Med. 2021 Nov 23;11(12):1241. doi: 10.3390/jpm11121241 (PMC8707113; doi:10.3390/jpm11121241)
Supplement: Supplementary file 1 [file jpm-11-01241-s001.zip › jpm-1446057-SI.pdf]

Supplemented data:

Table S1. Comparison of baseline criteria of patients with the worst cardiac function parameters [EF > 45% and CVP >10mm/Hg, (*n* = 61)] and 45 patients with the same demographics and clinical characteristics but better cardiac function (*n* = 45).

	**CVP>10mm/Hg and EF <45% (*n* = 61)**	**All others (*n* = 45)**	***P* value**
Age(years),median;IQR	72(63–83)	71 (61–81)	0.823
Family history,*n*(%)	2(3)	2(4.4)	0.561
Hypertension ,*n*(%)	36(59)	25 (55)	0.231
EGFR<60 ,*n*(%)	27 (44)	18(40)	0.119
Multivessel CAD,*n*(%)	38 (62)	23 (51)	0.06
Past MI, *n*(%)	13(21)	9(20)	0.562
Door to balloon, median;IQR	50(30–68)	45 (30–65)	0.167
Time to reperfusion, median, IQR	180(118–870)	160 (105–740)	0.07
Heart failure, *n*(%)	23(38)	14(31)	0.08
VT/VF, *n*(%)	19(31)	10(20)	0.04
Atrial fibrillation, *n*(%)	7(11)	5(11)	0.275
Mechanical ventilation *n*(%)	15(25)	7(16)	0.02
Acute kidney injury *n*(%)	29(48)	16(35)	0.05
